# The role of the UPS in cystic fibrosis

**DOI:** 10.1186/1471-2091-8-S1-S11

**Published:** 2007-11-22

**Authors:** Emma L Turnbull, Meredith FN Rosser, Douglas M Cyr

**Affiliations:** 1 Department of Cell and Developmental Biology, 526 Taylor Hall, Mason Farm Road, UNC-Chapel Hill School of Medicine, University of North Carolina, Chapel Hill, North Carolina 27599, USA

## Abstract

CF is an inherited autosomal recessive disease whose lethality arises from malfunction of CFTR, a single chloride (Cl^-^) ion channel protein. CF patients harbor mutations in the *CFTR* gene that lead to misfolding of the resulting CFTR protein, rendering it inactive and mislocalized. Hundreds of CF-related mutations have been identified, many of which abrogate CFTR folding in the endoplasmic reticulum (ER). More than 70% of patients harbor the ΔF508 CFTR mutation that causes misfolding of the CFTR proteins. Consequently, mutant CFTR is unable to reach the apical plasma membrane of epithelial cells that line the lungs and gut, and is instead targeted for degradation by the UPS. Proteins located in both the cytoplasm and ER membrane are believed to identify misfolded CFTR for UPS-mediated degradation. The aberrantly folded CFTR protein then undergoes polyubiquitylation, carried out by an E1-E2-E3 ubiquitin ligase system, leading to degradation by the 26S proteasome. This ubiquitin-dependent loss of misfolded CFTR protein can be inhibited by the application of ‘corrector’ drugs that aid CFTR folding, shielding it from the UPS machinery. Corrector molecules elevate cellular CFTR protein levels by protecting the protein from degradation and aiding folding, promoting its maturation and localization to the apical plasma membrane. Combinatory application of corrector drugs with activator molecules that enhance CFTR Cl^-^ ion channel activity offers significant potential for treatment of CF patients.

**Publication history:** Republished from Current BioData's Targeted Proteins database (TPdb; ).

## Introduction

One of the most common inherited genetic diseases is CF [1], which affects 1 in 3200 births globally, culminating in ~1000 new diagnoses annually. Due to the high frequency of CF, the gravity of the symptoms and the resulting mortality, it is imperative that research is carried out to gain a better understanding of the disease and to develop new therapies. CF manifests due to mutation(s) in the *CFTR* gene, whose protein product is a cAMP-regulated Cl^-^ ion channel belonging to the ATP binding cassette family [2]. In non-CF patients the CFTR protein is predominantly localized to the apical membrane of ciliate cells that line the lungs and gut, where it regulates Cl^-^ ion movement across epithelia [3,4]. CFTR mutations that abrogate channel function inhibit trans-epithelial ion transport, which in turn leads to onset of CF symptoms such as pancreatic failure and lung disease, the greatest cause of CF patient mortality [3,5].

## CFTR biogenesis

**Figure 1:**
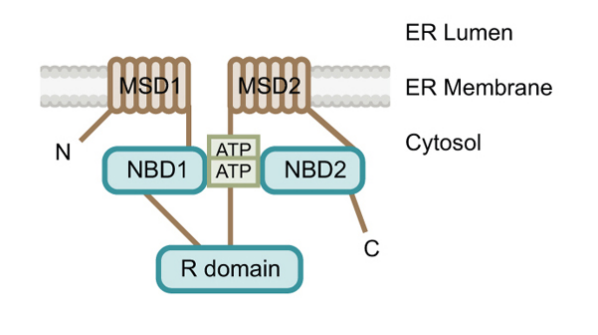
CFTR domain layout in the ER membrane lipid bilayer during ATP hydrolysis, depicting the membrane spanning domains (MSD), nucleotide binding domains (NBD) and regulatory domain (R). The membrane spanning domains are depicted in sepia and the cytosolic domains in aqua.

**Figure 2:**
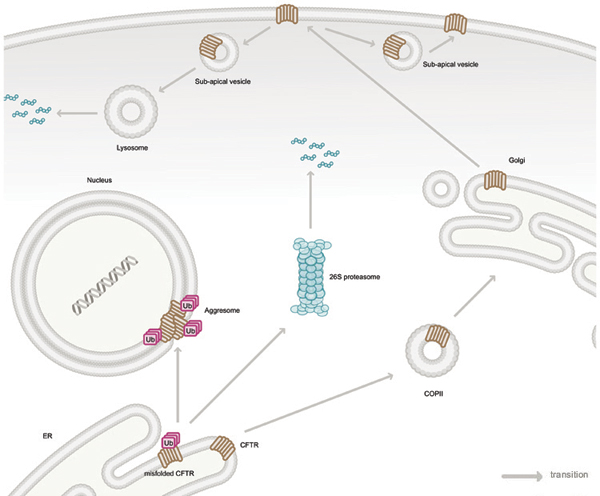
CFTR is co-translationally inserted into the ER membrane during ribosomal translation of CFTR mRNA from the nucleus. If CFTR is misfolded in the ER it is ubiquitylated and retrotranslocated to the cytosol, where it is degraded by the 26S proteasome. Upon inhibition of the proteasome, ubiquitylated CFTR is localized to a pericentriolar aggresome structure. Correctly folded CFTR proteins are transferred to the Golgi apparatus for glycolytic maturation via the coat complex II (COPII) machinery. Mature CFTR is exported to the plasma membrane to function as a chloride ion channel. CFTR protein levels at the plasma membrane are regulated by sub-apical vesicles delivering CFTR protein for either lysosomal degradation or recycling. For simplicity, we have represented CFTR with a single membrane spanning sepia symbol. Readers are invited to refer to figures 1 and 3 for the full domain architecture of the protein.

CFTR is a 1480 amino acid polytopic glycomembrane protein comprised of two membrane-spanning domains (MSD1 and MSD2) (each containing six transmembrane domains (TMD)), two cytoplasmic nucleotide binding domains (NBD1 and NBD2) and a regulatory (R) region [3] (Figure 1). CFTR folding occurs in the ER and necessitates that the cytoplasmic domains be folded properly to ensure intramolecular interaction between MSDI and MSDII [6], ultimately resulting in the NBDs and R region forming a functional ion channel [7-9]. The NBDs of CFTR are responsible for binding and hydrolyzing ATP to enable ion channel function [2]. The co-translational folding of CFTR (A-form) is an inefficient, slow and complex process [10,11] whereby the nascent polypeptide is concomitantly folded and inserted into the ER lipid bilayer [12] (Figure 2). Not surprisingly, ~55–80% of newly synthesized wild-type CFTR protein is improperly folded and targeted to the cytoplasmic proteasome for degradation in human cells [13-15], proposed to be due the complex and error prone folding process.

The folded and ER membrane-inserted CFTR, referred to as the immature B-form, leaves the ER via coat protein complex II (COPII)-coated vesicles [16,17] (Figure 2). CFTR then enters the Golgi apparatus where two of the Asn-linked glycans in the fourth extracellular loop are converted from immature high-mannose forms to mature complex oligosaccharides, creating C-form CFTR [12]. The mature CFTR protein is subsequently delivered to the plasma membrane where it functions as a Cl^-^ ion channel. At the apical membrane, CFTR levels are regulated by sub-apical vesicle internalization, resulting in one of two fates: recycling to the plasma membrane or lysosomal degradation [12].

## CFTR mutations

Over a thousand CF disease-related mutations have been identified to date, which yield a wide of range of defects in the CFTR protein. CF disease-related mutations are assigned into different classes depending on their molecular characteristics [18,19]. Class I mutants include deletions, frameshifts and non-sense mutations that result in prematurely truncated CFTR protein products, class II mutants are defective in intracellular trafficking (although they may exhibit a level of ion channel activity) and class III mutants are full-length proteins with little or no ion channel activity. Class IV mutants generally result in a less severe phenotype, as the CFTR protein only exhibits slightly reduced channel activity. Class V mutants proteins are functional but expressed at reduced levels, while class VI mutants are expressed at wild-type levels but exhibit decreased stability at the plasma membrane. The more severe CF symptoms are associated with class I, II and III mutations due to the almost complete absence of channel activity at the plasma membrane [18,19].

F508 CFTR, a temperature-sensitive class II mutation, is the most commonly identified mutation in CF patients, accounting for 70% of CF mutant alleles [20]. Other identified class II disease-causing mutations in CFTR include N1303K, G85E and G91R [18]. These class II mutations all result in a misfolded CFTR protein that is recognized by the quality control machinery and thus prematurely degraded. The exact mechanism by which these mutations disrupt folding is not completely clear [21], but both the G85E and G91R mutations have been shown to affect folding due to the insertion of a charged residue in the plane of the lipid bilayer [9].

## The ubiquitin proteasome system (UPS)

A cell's necessity to remove and degrade misfolded proteins directly results in the sorting of these proteins, diverting them from a folding to a degradation pathway. In addition, selecting substrates for degradation prevents their accumulation into insoluble and potentially toxic aggregates. Degradation of the misfolded proteins is carried out by the UPS, whereby substrates are polyubiquitylated and then degraded by the cytosolic proteasome [22-25]. Ubiquitylation refers to the addition of ubiquitin, a small monomeric 76 amino acid polypeptide, by covalent linkage to lysine residues on the substrate molecules [26]. Ubiquitylation is a multistep process involving three classes of enzymes: E1 ubiquitin activating enzymes, E2 ubiquitin conjugating (UBC) enzymes and E3 ubiquitin protein ligases (See [27] for review). The ubiquitin process is initiated through activation of an E1 by hydrolysis of ATP to promote the formation of a thioester bond between an internal active site cysteine and the C-terminal glycine of ubiquitin [26]. The activated ubiquitin is then transferred to the E2 active site cysteine where a new thioester linkage is formed [26]. Finally, the activated ubiquitin is covalently attached by an E3 ligase to the ε-amino group of a lysine side chain on the substrate protein [26]. Depending on the type of E3 involved, this step can entail an initial transfer of ubiquitin from the E2 to the E3 or the E3 can catalyze the transfer of ubiquitin directly from the E2 to the substrate protein [28]. Once a single ubiquitin molecule has been conjugated to a substrate, polyubiquitylation can occur by linking single ubiquitin molecules together to form a ubiquitin chain. Ubiquitin contains numerous lysine residues, any of which has the capacity to form isopeptide bonds. Interestingly, chains linked at lysine-63 are not targeted for degradation, whereas those linked by lysine-29 and lysine-48 are [29]. Ubiquitin chains vary in length and linkage [30], and their addition results in a variety of outcomes [31]. However, only polyubiquitylated proteins are targeted for 26S proteasomal degradation [29].

In the case of ER-localized proteins, polyubiquitylated substrates are dislocated from the ER membrane to enable translocation to the cytoplasmic 26S proteasome, where they are selectively degraded [24,29]. The proteasome is a large 2.5 MDa multi-subunit complex comprising around 30 subunits [29] that degrades substrates with four or more ubiquitin molecules, with a tetra-ubiquitin motif being the minimum requirement for efficient proteasomal targeting [32].

## CFTR and the UPS

**Figure 3:**
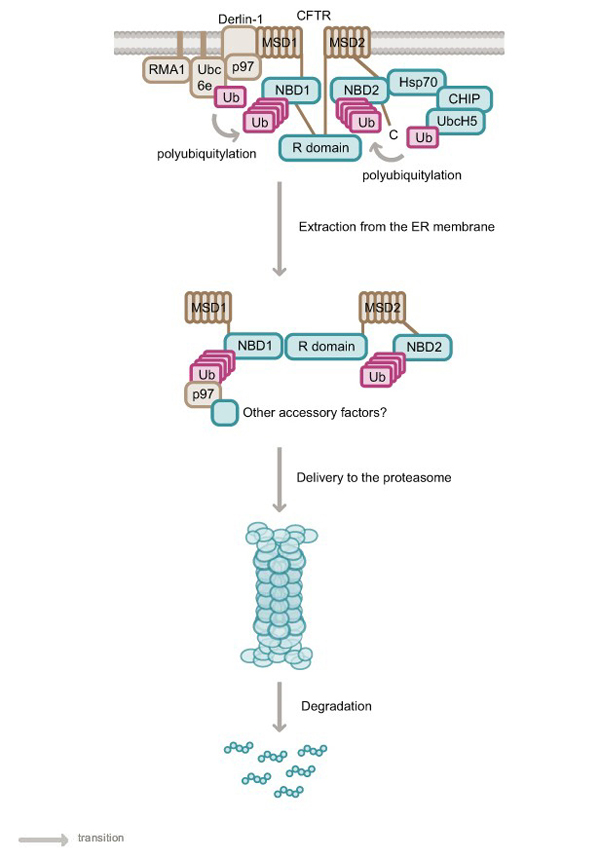
The UPS complexes located in the ER membrane (Derlin-1, RMA1, Ubc6e and p97) and cytoplasm (Hsp70 and CHIP) are shown. The figure demonstrates the polyubiquitylation of CFTR due to membrane-bound and cytosolic E3 ubiquitin ligase complexes. While the ubiquitylation likely occurs on the cytoplasmic domains of CFTR, the exact lysine residues conjugated with ubiquitin are unknown. p97 and possibly other associated factors are thought to participate in the extraction and delivery of CFTR from the ER membrane to the cytosolic proteasome. However, it is unknown whether the CFTR protein is extracted from the membrane in one piece, or degraded into smaller domains before retrotranslocation. Membrane spanning domains are depicted in sepia and the cytosolic domains in aqua.

Misassembled CFTR mutants appear to be detected during the folding process via two distinct systems in human cells; one that senses defects located within the cytoplasm [21,33,34] and the other within the ER membrane [35] (Figure 3). Distinct complexes of ubiquitylating proteins act in conjunction with factors such as Hsp70 and Derlin-1, which appear to recognize misassembled substrates at CFTR folding checkpoints in the cytosol and within the ER membrane, respectively.

CFTR proteins with mutations that cause misfolding of their cytoplasmic regions, (NBD1, NBD2 and the R domain) are detected by the cytosolic chaperone Hsp70. Hsp70 is believed to maintain the misfolded substrate in a soluble state, and upon interaction with the E3 ubiquitin ligase CHIP, the Hsp70–CHIP complex diverts CFTR from the folding to the degradation pathway [33,34,36-38]. CHIP promotes ubiquitylation and degradation of CFTR in association with the cytosolic E2 ubiquitin conjugating enzyme UbcH5a [33,34]. CHIP carries out this function by mediating the attachment of ubiquitin to a chaperone-presented CFTR, thereby stimulating its proteasomal degradation [33,34,36-38]. The role of CHIP in the ubiquitylation and degradation of CFTR has been demonstrated both *in vitro* by reconstitution of the ubiquitylation reaction [34], and in human cells by overexpression and pulse chase analysis [33].

The important role of CHIP in the degradation of CFTR is evident in studies in which CHIP's activity is inhibited by one means or another. for example, CHIP's E3 ubiquitin ligase activity can be regulated by other Hsp70 co-chaperones such as BAG-2 and HspBP1 [39-41]. BAG-2 inhibits the ubiquitin ligase activity of CHIP by abrogating the CHIP–E2 cooperation and stimulates the chaperone-assisted maturation of CFTR [40,41]. Likewise, overexpression of HspBP1, which has been shown to inhibit CHIP's activity and is a nucleotide exchange factor that can promote the release of substrates from Hsp70, stimulates the maturation of CFTR [39]. Furthermore, inhibition of the CHIP–Hsp70 E3 ubiquitin ligase complex by overexpression of a CHIP mutant results in the accumulation of a folding-competent stable B-form of CFTR [34]. Additionally, geldanamycin treatment of microsomes containing *in vitro* translated CFTR results in the release of the CFTR protein from Hsp70, which coincides with the cessation of ubiquitylation and formation of stable B-form CFTR [42], which is resistant to ubiquitylation/degradation [13,14,43].

However, while Hsp70 is necessary for the degradation of CFTR by CHIP, it also plays an important role in the folding pathway of CFTR [7,44]. This idea is supported by both *in vitro* studies in which Hsp70 can prevent the aggregation of the NBD1 domain [7], and cell culture studies which show that the induction of Hsp70 results in the increased trafficking of mutant ΔF508 CFTR to the plasma membrane [44]. There must exist a yet unexplained mechanism that determines whether Hsp70-bound substrates are allowed to fold or be targeted for degradation by the co-chaperone, CHIP.

In addition to the CHIP–Hsp70 complex, which monitors the cytosolic domains of CFTR, there are also membrane-anchored proteins that can potentially monitor the assembly of CFTR membrane domains. Aberrant CFTR folding within the ER lipid bilayer is proposed to be identified by the ER quality control (QC) factor Derlin-1 and its associated proteins [35,45]. Co-immunoprecipitation analysis of both yeast and human cells has shown that Derlin-1 associates with substrate proteins and other QC factors such as the retro-translocation factors p97/Cdc48 and VIMP, E2 (Ubc6e) and E3 ubiquitin ligases (RMA1, HRD1 and gp78), and the deglycosylating enzyme peptide *N*-glycanase [35,45-51]. Studies in human cells have shown that the overexpression of Derlin-1 leads to the retention of CFTR in the ER [35,45], while RNAi mediated knockdown of Derlin-1 leads to an increase in steady state levels of mutant CFTR [45]. These results suggest that Derlin-1 can participate in the selection of misfolded membrane proteins such as CFTR for ER-associated degradation (ERAD).

Within the context of the ER membrane, overexpression studies in human cells have shown that the E2 ubiquitin conjugating enzyme Ubc6e functions in association with the E3 ubiquitin ligase RMA1 to mediate the ubiquitylation of aberrant CFTR proteins, promoting their degradation. Both Ubc6e and RMA1 are localized to the cytosolic face of the ER membrane via their C-terminal domains [35,52-54] and therefore are likely to ubiquitylate cytosolic regions of CFTR that are exposed in its misfolded form. while both of these proteins have been isolated in complex with Derlin-1 [35], it has not been established whether or not this interaction is necessary for Ubc6e and RMA1 to promote the ubiquitylation of CFTR.

Misfolded membrane proteins that are identified by the ER QC factors are polyubiquitylated and dislocated from the ER membrane, then transported to the 26S proteasome for degradation. Yeast lacking Derlin-1 show increased stability and accumulation of ERAD substrate protein in the ER lumen, suggesting an additional function for Derlin-1 in retro-translocation [55]. Derlin-1 appears to contribute to retro-translocation of substrates such as MHC class I heavy chain molecules from the ER to the cytosol in association with p97 [46,48,56]. p97 is also required for mutant CFTR degradation [57,58] and specifically associates with ubiquitylated CFTR proteins [45]. It acts to remove ubiquitylated CFTR from the ER to enable its degradation [59], as disruption of p97–CFTR complexes results in accumulation of immature CFTR in the ER [60,61].

## CF disease models, knockouts and assays

Basic CF research to understand CFTR biogenesis and degradation has been carried out in immortalized cell line systems such as HEK293 transfected with *CFTR* due to ease of manipulation [62]. Theses findings have subsequently been investigated in *CFTR*-expressing cell lines such as Fischer rat thyroid (FRT) epithelial cells [63], and in primary mouse cultures of polarized epithelial cells [64], both of which represent more biologically relevant model systems. To extend the observations made in transformed and rodent model cell lines, human bronchial epithelial (HBE) cells cultured as a monolayer have also been employed [65]. HBE cells are not used in initial studies as they are less amenable to manipulation by transfection and *CFTR* expression is low. However, HBE cells make excellent candidates for testing potential drug treatments.

There have been multiple attempts to create mouse models of CF that mimic the human disease (see [66] for comprehensive review). Unfortunately, there are limitations in using whole organisms such as mice for physiological studies due to differences in airway epithelial biology when compared with humans that present challenges in evaluating CF therapies using murine models [67]. Nevertheless, the models that would be of the greatest use for studies relating to the role of the UPS in CF are ΔF508 models. Three such models have been generated by Doorninck *et al.*[68], Colledge *et al.*[69] and Zeiher and colleagues [70]. The models show different levels of survival and different phenotypes, likely due to the differences in mRNA and proteins levels. To date, due to the difficulties in performing *in vivo* studies on protein folding and degradation, these models have mainly been used to study the physiological effects of the ΔF508 mutant. However, ΔF508 CFTR exhibits a temperature sensitive folding and processing defect in epithelial cells isolated from the ΔF508 CFTR mouse [69]. Therefore, these models should prove useful in the study of the role of the UPS in CF and for the development of novel therapeutic approaches based on overcoming the folding and processing defect. As the list of QC factors that participate in the degradation of mutant CFTR is further developed, the CF field can also benefit from studies in which the ΔF508 mouse models are crossed with knockout models of the different QC factors.

## Drugs with potential for CF therapeutics

Restoration of just 5% of wild-type CFTR function dramatically improves lung and gut function in CF patients [71]. Research efforts into CF therapy development have generated drugs that can be divided into the different classes of potentiators or correctors, both of which will be discussed in more detail. In addition, the possibility of developing compounds that activate alternative Cl^-^ and K^+^ channels to compensate for the loss of CFTR activity is promising according to studies in both HEK 293 cells and IB3-1 cells, which were isolated from a CF patient expressing the ΔF508 mutation. In these studies, the cells show an increase in Cl^-^ transport due to manipulation of ClC-2 channels through extracellular pH or by arachidonic acid, amidation or acid-activated omeprazole [72,73].

Potentiator drugs act to open up the malfunctioning CFTR channel, thereby promoting better ion and fluid trafficking through epithelia to relieve CF patient symptoms. This class of drugs is important for those CF mutations that result in reduced channel activity, but potentiation of channel activity alone does not solve the trafficking problems seen with the class II mutants such as ΔF508 CFTR. Alternatively, corrector molecules can help correct the folding and trafficking defect of mutant CFTRs, but they do not necessarily solve the problems of low channel activity or stability at the plasma membrane. Therefore, mutations such as F508, which exhibit defects in both folding and channel activity, will likely require combinatorial drug approaches.

Genistein [74-77] is a molecule that has been widely used in research assays as a potentiator of CFTR channel activity. It is a tyrosine kinase inhibitor but may act by binding directly to the NBDs of CFTR and stabilizing their dimerization [78]. However, like other potentiators, genistein has been shown to have a dual effect where low micromolar concentrations stimulate CFTR chloride currents, but higher concentrations inhibit CFTR channel activity [79]. CFpot-532 is another potentiator that was recently identified by Vertex Pharmaceuticals [80,81]; however, the mechanism of action for this drug is still unknown. The ability of CFpot-532 to act as a potentiator for mutant CFTR channels was first demonstrated with temperature-corrected ΔF508 CFTR expressed in NIH 3T3 cells [81], and was later confirmed using low temperature rescue in BHK cells stably expressing ΔF508 CFTR [80]. This drug has exciting possibilities since studies in the BHK cells showed that it could also act as a specific corrector for CFTR and promote the maturation and trafficking of ΔF508 CFTR (but not that of a mutant P-glycoprotein) to the plasma membrane [80].

Corrector compounds aid CFTR folding [82], shielding misfolded CFTR from the UPS, which results in a greater level of CFTR protein and increased potential that it will reach the plasma membrane and provide ion channel activity. Although misfolded, certain CFTR mutants, such as F508, have residual channel activity if helped to reach the plasma membrane. It is unclear how corrector molecules function *in vivo* and further investigations are required to elucidate their modes of action.

Several classes of small molecule correctors have been identified, including curcumin [83], compound 9 [84], VRT-325 [81], CFpot-532 [80], Corr-3a and Corr-4a [82]. Curcumin is an ingredient in curry spice that acts as a calcium-adenosine triphosphatase pump inhibitor, and was initially identified as a compound that could correct the folding defect of ΔF508 CFTR in mice [83]. Though curcumin has been tested in phase I clinical trials, there is some controversy surrounding whether it acts as a corrector for CFTR in biological systems, as these initial results have not been reproduced by many labs [85-87]. Furthermore, both curcumin and compound 9 have been reported to be inactive in ΔF508 HBE cells [81], making them unlikely therapy candidates for CF patients. The fact that these drugs were initially identified in model experimental systems, and that results could not be recapitulated in more relevant systems such as HBE cells and human patients, underscores the difficulty of developing small molecule therapeutics for CF. In terms of the current potential drug therapies for CF patients, VRT-325 (developed by Vertex Pharmaceuticals) [81], Corr-3a and Corr-4a (developed by Verkman and colleagues at University of California, San Francisco) [82] are excellent candidates. They were identified by high-throughput screens and function to increase CFTR protein levels and elevate ion channel activity. However, these drugs are still in the pre-clinical stage, and the exact mechanisms by which they function are not yet elucidated.

Application of Corr-4a or VRT-325 molecules promotes increased protein levels of ΔF508 CFTR at the plasma membrane [81,82]. This is presumably because these correctors increase folding efficiency post-translationally, as VRT-325 promoted correct folding of mutant CFTR TMDs [88], thereby reducing ERAD and resulting in greater export of mutant CFTR to the cell surface [81,82]. It is not known if these compounds act directly with the CFTR protein to correct a folding defect, or if they act indirectly through other proteins or perhaps through modification of the lipid bilayer itself. However, it is known that none of these corrector molecules affect CFTR translation [81,82] and that VRT-325 does not inhibit the UPS [81]. ΔF508 CFTR channel activity was increased by treatment with VRT-325 in HBE cells from CF patients and Corr-4a and Corr-3a at 37°C in human airway epithelial [81,82]. However, Corr-3a was unable to sustain ΔF508 CFTR ion channel function for longer than 24 hours [82]. The inability of theses correctors to sustain CFTR channel activity in the long term needs to be considered in terms of their potential for patient treatment. Promisingly, the activities of ‘Corr’ correctors are specific for ΔF508 CFTR in HBE cells and did not affect the CFTR mutants P574H or N1303K, or the dopamine receptor mutant [82]. However, the activities of VRT-325 are not specific for CFTR, as these molecules also increased cell surface expression of the cardiac potassium channel hERG mutant G601S, which causes hereditary human long-QT syndrome type 2 [81]. Overall, the level of correction achieved with these molecules is currently low, and alternative molecules or combinations need to be developed that correct CFTR folding and activity more efficiently and effectively.

## Next frontiers

Combinations of molecules for CF treatment are likely to be the most promising method of elevating CFTR protein levels and increasing ion channel activity. For example, the potentiator VRT-532 was able to potentiate ion channel activity in the CFTR mutants ΔF508 and G551D, inferring its potential as a drug treatment for CF patients in combination with VRT-325 [81]. In fact, in primary homozygous ΔF508 CF airway cultures, treatment with both VRT-325 and VRT-532 increased ΔF508 CFTR maturation and resultant channel activity to levels greater than observed for each molecule independently [81].

With the identification of new CFTR degradation-associated QC factors, targeting the UPS system and enhancing ion channel activity of misfolded mutant CFTR in parallel is an optimistic avenue for current CF research. Further investigations are required to develop molecules that are specific to CFTR and that can sustain their additive effect on the patient. Many considerations must be taken into account during the development of new therapeutic molecules, such as the longevity of their effect, the effectiveness of their abilities and the specificity of their activity towards CFTR. The combination of new insights into CFTR QC factors and the UPS, and the rapid development of CF drug molecules such as correctors, has the potential to create therapies that will benefit CF patients.

## Abbreviations

CF = Cystic Fibrosis; CFTR = Cystic Fibrosis Transmembrane Receptor; COPII = Coat Complex II; ER = Endoplasmic Reticulum; ERAD = ER-associated degradation; FRT = Fischer Rat Thyroid; HBE = Human Bronchial Epithelia; MSD = Membrane Spanning Domain; NBD = Nucleotide Binding Domain; QC = Quality Control; R = Regulatory; TMD = Transmembrane Domains; UBC = Ubiquitin conjugating ; UPS = Ubiquitin Proteasome System.

## Competing interests

The authors declare that they have no competing interests.

## Publication history

Republished from Current BioData's Targeted Proteins database (TPdb; ).
